# Significance of E-cadherin expression in triple-negative breast cancer

**DOI:** 10.1038/sj.bjc.6605735

**Published:** 2010-06-15

**Authors:** S Kashiwagi, M Yashiro, T Takashima, S Nomura, S Noda, H Kawajiri, T Ishikawa, K Wakasa, K Hirakawa

**Affiliations:** 1 Department of Surgical Oncology, Osaka City University Graduate School of Medicine, 1-4-3 Asahi-machi, Abeno-ku, Osaka, Japan; 2 Oncology Institute of Geriatrics and Medical Science, Osaka City University Graduate School of Medicine, 1-4-3 Asahi-machi, Abeno-ku, Osaka, Japan; 3 Department of Diagnostic Pathology, Osaka City University Graduate School of Medicine, 1-4-3 Asahi-machi, Abeno-ku, Osaka, Japan

**Keywords:** triple-negative breast cancer, E-cadherin, prognostic marker, intrinsic subtype, breast cancer

## Abstract

**Purpose:**

Triple-negative breast cancer (TNBC), a subtype of breast cancer that is oestrogen receptor (ER) negative, progesterone receptor (PR) negative, and human epidermal growth factor receptor 2 (HER2) negative, has a poor prognosis. Although a correlation between E-cadherin expression level and outcome has been demonstrated among all types of breast cancer, little is known about the significance of E-cadherin expression levels in TNBC.

**Methods:**

A total of 574 patients who had undergone a resection of a primary breast cancer except for invasive lobular carcinomas were enrolled in this study. Expressions of ER, PR, HER2, and E-cadherin were assessed by immunohistochemistry. We examined the association between TNBC and other clinicopathological variables and evaluated the significance of the E-cadherin expression.

**Results:**

Among the 574 breast cancer cases, 123 (21.4%) revealed a triple-negative phenotype. Patients with TNBC experienced more frequent lymph node metastasis (*P*=0.024) and a poorer prognosis (*P*<0.001) in comparison with non-TNBC patients. Triple-negative breast cancer was an independent prognostic factor. Reduced levels of E-cadherin were observed in 238 (41.5%) of the 574 breast cancer cases. E-cadherin reduction was significantly frequent in cases of TNBC (*P*<0.001) and lymph node metastasis (*P*=0.032). Furthermore, in the 123 TNBC cases, the prognosis of patients with an E-cadherin-negative expression was significantly worse than that of E-cadherin-positive patients (*P*=0.0265), especially for those in clinical stage II (*P*=0.002). A multivariate logistic regression analysis showed a reduction of the E-cadherin expression to be an independent prognostic factor (*P*=0.046).

**Conclusion:**

E-cadherin expression may be a useful prognostic marker for classifying subgroups of TNBC.

Human breast cancers represent a heterogeneous group of tumours that display significant diversity with regard to clinical behaviour, outcome, and response to therapy (Perou *et al*, 2000; Abd El-Rehim *et al*, 2005; Mattie *et al*, 2006). The prognosis and management of breast cancer is influenced by the status of oestrogen receptor (ER), progesterone receptor (PR), and human epidermal growth factor receptor 2 (HER2) of the tumour. The mortality of breast carcinoma is decreasing because of recent developments in diagnostic techniques and therapies; however, the mortality of triple-negative breast cancer (TNBC), a subtype of breast cancer that is ER negative, PR negative, and HER2 negative, remains high (Sorlie *et al*, 2001, 2003; Rakha *et al*, 2006; Bauer *et al*, 2007). In addition to being a clinically heterogeneous disease, breast cancer is also molecularly heterogeneous. Breast cancer subgroups are primarily defined by ER and HER2 expression, although the different prognostic signatures have not yet been clearly evaluated in these different molecular subgroups (Derksen *et al*, 2006). As molecular biological characterisation of these groups is still uncertain, the characterisation of TNBC may be important for evaluating patients’ outcomes and for developing a molecular-based medicine treatment strategy.

E-cadherin is a calcium-regulated homophilic cell–cell adhesion molecule. E-cadherin inactivation is one of the changes that characterise the invasive breast cancer phenotype. Transfection of cDNA encoding E-cadherin into highly invasive mouse mammary tumour cell lines resulted in decreased invasiveness and metastasis (Meiners *et al*, 1998). Previous studies have shown correlations between decreased levels of E-cadherin expression and distant metastases, as well as patient outcomes (Siitonen *et al*, 1996; Charpin *et al*, 1998; Heimann *et al*, 2000). However, little is known about the E-cadherin expression levels of TNBC. To reduce mortality from TNBC, there is a need to examine and characterise tumours of poor prognosis, to predict their biology, to ensure adequate therapy, and to improve patients’ outcome (Mahler-Araujo *et al*, 2008). In this study, we classified 123 cases of breast cancer with the triple-negative phenotype from 574 breast carcinomas. We addressed the significance of clinicopathological features and E-cadherin expression to identify additional prognostic markers that can identify tumours with more aggressive behaviour.

## Materials and methods

### Patients

This study investigated a consecutive series of 574 cases of sporadic invasive breast carcinoma, except for invasive lobular carcinomas. All patients underwent a curative operation of a mastectomy or a conservative surgery with axillary lymph node dissection at our department from 2000 to 2006. All of the patients who had undergone conservative breast surgery received postoperative radiotherapy to the residual breast. Each patient was treated with suitable adjuvant therapy postoperatively according to the stage of the disease. This study was approved by the Osaka City University ethics committee. Informed consent was obtained from all patients before entry. The disease-free interval was defined as the interval in months, from the date of the primary surgery to the first local recurrence or distant metastasis. The overall survival was the time, in weeks, from the date of the primary surgery to the time of breast cancer-related death. Tumours were confirmed histopathologically and staged according to the TNM classification (Singletary and Greene, 2003).

### Immunohistochemistry

**Figure 1:**
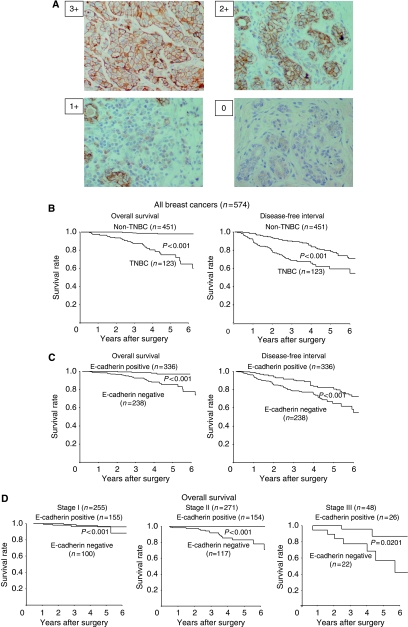
(**A**) Immunohistochemical determination of E-cadherin. (**B**) Correlation between triple-negative phenotype and overall survival or disease-free interval in the breast cancer series. (**C**) Probability of survival of breast cancer patients in relation to the E-cadherin expression. (**D**) The overall survival of patients with resectable breast cancer according to the clinical stage.

All tissues were fixed in 10% neutral-buffered formalin immediately after surgical resection and embedded in paraffin using standard protocols (Meiners *et al*, 1998; Mattie *et al*, 2006). In brief, the slides were deparaffinised and were heated for 20 min at 105°C by autoclave in Target Retrieval Solution (Dako, Carpinteria, CA, USA). Sections were then incubated with 3% hydrogen peroxide to block endogenous peroxidase activity. Thereafter, sections were incubated in 10% normal goat or rabbit serum to reduce non-specific antibody binding. Primary monoclonal antibodies were directed against ER (clone 1D5, dilution 1 : 80; Dako, Cambridge, UK), PR (clone PgR636, dilution 1 : 100; Dako), HER2 (Hercep Test, Dako), and E-cadherin (clone NCH-38, dilution 1 : 200; Dako). Tissue sections were incubated with each antibody for 70 min at room temperature or overnight at 4°C. After washing in phosphate-buffered saline (PBS), tissues were incubated with horseradish peroxidase-conjugated anti-rabbit or anti-mouse Ig polymer as a second antibody (Envision kit, Dako) for 30 min at room temperature, according to the manufacturer's instructions. The slides were treated with streptavidin–peroxidase reagent, and were incubated in PBS diaminobenzidine and 1% hydrogen peroxide v/v, followed by counterstaining with Mayer's haematoxylin. Positive and negative controls for each marker were used according to the supplier’s data sheet (Dako). Immunohistochemical scoring was performed in a blind manner. The cutoff for ER positivity and PR positivity was ⩾10% positive tumour cells with nuclear staining. Human epidermal growth factor receptor 2 positive was defined as either HER2 gene amplification or scored as 3+. E-cadherin antibody stained the membrane intensely and the cytoplasm of cancer cells weakly. E-cadherin expression was semi-quantitatively analysed according to the percentage of cells showing membrane positivity: 0, 0–10% 1+, 10–30% 2+, 30–70% 3+, >70%. E-cadherin expression was considered positive when scores were ⩾2, and negative when scores were ⩽1 (Figure 1A). A case with cytoplasmic staining only was determined as E-cadherin negative.

### Statistical analysis

Statistical analysis was performed using SPSS 13.0 statistical software (SPSS Inc., Chicago, IL, USA). We examined the association between TNBC and other clinicopathological variables, and the significance of different prognostic markers using *χ*^2^ test, and *χ*^2^ test for trend as appropriate. The association with survival was analysed initially by Kaplan–Meier plot and log-rank test and also with Cox regression analysis to adjust for other prognostic indicators. A *P*-value of <0.05 was considered significant. Cutoff values for different biomarkers included in this study were chosen before statistical analysis.

## Results

### Identification and clinicopathological features of 123 TNBCs

In this study, 574 cases of breast carcinomas were analysed for the three markers (namely ER, PR, and HER2). Of these cases, 123 (21.4%) showed a triple-negative phenotype (ER negative, PR negative, and HER2 negative). Table 1 shows the clinicopathological features of TNBC, as compared with those of non-TNBC. The 123 patients with TNBC had a median age of 58 years (range, 26–93 years). A statistically significant difference was observed between TNBC and non-TNBC with regard to the degree of pathological stage (*P*=0.005), tumour size (*P*=0.012), lymph node metastasis (*P*=0.024), and lymphatic invasion (*P*=0.015). The median overall survival of the 574 patients was 45.7 months (range, 5.8–72 months), and the median time of disease-free interval was 42.5 months (range, 4.8–72 months). Patients with TNBC experienced significantly poorer outcomes in terms of overall survival (*P*<0.001, log rank) and disease-free interval (*P*<0.001, log rank) in comparison with patients with non-TNBC (Figure 1B). A univariate analysis of the 574 breast cancer cases demonstrated significant correlations between overall survival and reduction in E-cadherin (*P*<0.001), TNBC (*P*<0.001), cancer stage (*P*=0.049), lymph node disease (*P*=0.001), and lymphatic invasion (*P*=0.040). On the basis of a multivariate logistic regression analysis of the tumour stage, lymph node status, lymphatic invasion, TNBC, and E-cadherin expression, the TNBC subtype was the only variable of independent prognostic significance in the 574 breast cancer cases (Table 2).

### E-cadherin expression in breast cancer

**Figure 2:**
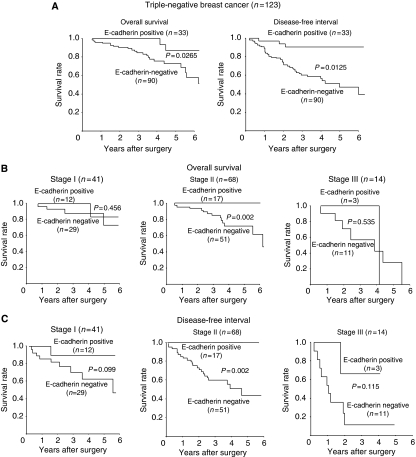
Survival of patients with triple-negative breast cancer. (**A**) Correlation between the E-cadherin expression and either the overall survival or disease-free interval in the triple-negative phenotype. (**B**, **C**) The overall survival or disease-free interval according to the clinical stage.

Reduced E-cadherin expression was observed in 238 (41.5%) of the 574 breast cancer patients (Table 3). The reduction in E-cadherin was significantly frequent in cases of TNBC (*P*<0.001) and lymph node metastasis (*P*=0.032). E-cadherin-negative patients experienced significantly poorer outcomes in terms of overall survival (*P*<0.001, log rank) and disease-free interval (*P*<0.001, log rank) in comparison with patients who were E-cadherin positive (Figure 1C). Our findings indicated that the prognosis of E-cadherin-negative cases was significantly poorer than that of E-cadherin-positive cases at stage I, II, and III (Figure 1D). Although no significant association between E-cadherin expression and clinicopathological parameters was identified in the 123 TNBC cases (Table 3), TNBC patients with E-cadherin-negative expression experienced significantly poorer outcomes in terms of overall survival (*P*=0.0265, log rank) and disease-free interval (*P*=0.0125, log rank) than did E-cadherin-positive patients (Figure 2A). With regard to the clinical stage, the prognosis of E-cadherin-negative cancer patients was significantly poorer than that of E-cadherin-positive cancer patients with regard to overall survival (*P*=0.002) and disease-free interval (*P*=0.002) in stage II (Figures 2B and C). The disease-free interval in E-cadherin-negative cancer patients was shorter than that of E-cadherin-positive cancer patients in stage I and III, although no significant difference in prognosis was observed between the two groups. On the basis of a univariate analysis of 123 TNBC cases, overall survival significantly correlated with E-cadherin expression (*P*=0.028) and lymph node metastasis (*P*=0.026). A multivariate logistic regression analysis of the 123 TNBC cases also showed that the reduction of E-cadherin expression significantly correlated with overall survival (*P*=0.046), thus suggesting that E-cadherin is an independent prognostic factor for TNBC (Table 4).

## Discussion

The frequency of TNBC is reported to be 15–26% of all breast cancers (Carey *et al*, 2006; Rakha *et al*, 2007). Among the 574 breast cancer cases in this study, we identified 123 (21.4%) patients with the triple-negative phenotype. These TNBC patients showed frequent lymph node metastasis and lymphatic invasion and experienced significantly poorer outcomes in comparison with non-TNBC patients. On the basis of multivariate logistic regression analysis of the 574 breast cancer cases, the TNBC subtype was demonstrated to be an independent prognostic factor. These findings confirmed that TNBC has high biological malignancy, as previously reported (Sorlie *et al*, 2001, 2003; Rakha *et al*, 2007).

A reduction of E-cadherin expression was associated with poor outcome (*P*<0.001) and lymph node metastasis (*P*=0.032). This finding suggested that the decrease of E-cadherin expression might be linked to the development of lymph node metastases in breast cancer. Although some studies have shown a correlation between E-cadherin expression and outcome in breast cancer (Lipponen *et al*, 1994; Siitonen *et al*, 1996), Rakha *et al* (2007) observed no correlation between survival rates and E-cadherin expression. Differences in antibodies, cutoff values, or race are possible reasons for discrepancies among these studies. In this study, E-cadherin antibody was clearly localised at the cell–cell boundaries. Genetic or epigenetic alterations are reported to be one of the reasons for the reduction of E-cadherin expression. Sporadic breast cancer is reported to show a frequent loss of heterozygosity at 16q22.1 wherein E-cadherin is located (Cleton-Jansen *et al*, 1994). In addition, other studies have reported that repression of E-cadherin transcription preceded the subsequent acquisition of methylated CpG sites (Graff *et al*, 1995; Baranwal and Alahari, 2009). Loss of heterozygosity or promoter methylation at the E-cadherin locus might be responsible for the decrease of E-cadherin expression in breast cancer patients.

E-cadherin-positive cases at stage II have better prognosis than those at stage I. Out of 155 E-cadherin-positive patients at stage I, 3 were dead because of lung metastasis, whereas no patient with E-cadherin-positive tumours at stage II was dead. Such three patients did not receive any adjuvant chemotherapy. Adjuvant chemotherapy might be necessary for breast cancer patients at stage I. The number of patients with stage III disease might be insufficient for the estimation of statistical difference in this study, large numbers of patients with stage III disease might be necessary in future to conclude the significance of E-cadherin in patients with stage III disease.

In the 123 TNBC cases in this study, the prognosis of patients with E-cadherin-negative expression was significantly worse than that of E-cadherin-positive patients, especially in cases of stage II TNBC. As TNBC is a heterogeneous group of breast cancers, the clinical course of TNBC patients remains difficult to predict (Alizadeh *et al*, 2001). Therefore, additional markers are being studied to further refine disease classification, especially in patient subgroups the outcome of which cannot be accurately predicted using conventional parameters. Although E-cadherin failed to emerge as a prognostic factor in the 574 breast cancer cases, it was successful in identifying a poor prognosis subgroup of TNBC patients with E-cadherin expression. Our study showed that the reduction of E-cadherin expression was an independent prognostic factor in TNBC. These findings suggest that E-cadherin might be a useful predictive marker to classify prognostic subgroups of TNBC and to better understand these tumours. A close correlation between lymph node metastasis and E-cadherin dysfunction has been reported in various types of carcinomas. The frequent lymph node metastasis associated with TNBC might be explained by the loss of cell–cell adhesion due to E-cadherin dysfunction. The association between E-cadherin and ER promoter methylation has been previously reported in human breast tumours and correlates with clinical parameters (Graff *et al*, 1995; Li *et al*, 2006; Baranwal and Alahari, 2009). Promoter methylation may explain the correlation between the reduction of E-cadherin expression and ER-negative breast cancer.

In conclusion, 123 cases of TNBCs among 574 breast cancer patients showed a poor prognosis, and TNBC was demonstrated to be an independent prognostic factor. Furthermore, reduction of E-cadherin expression was an independent prognostic factor for TNBC. E-cadherin might be a useful predictive marker to classify prognostic subgroups of TNBC and to better understand these tumours.

## Figures and Tables

**Table 1:** Clinicopathologic feature of 123 triple-negative breast cancers in 574 breast cancers

**Parameters**	**TNBC (*n*=123)**	**non-TNBC (*n*=451)**	***P*-value**
*Age at operation (years)*			
⩽55	58 (22.7%)	198 (77.3%)	0.520
>55	65 (20.4%)	253 (79.6 %)	
			
*Pathological stage*			
I	41 (16.1%)	214 (83.9%)	0.005
II and III	82 (25.7%)	237 (74.3%)	
			
*pTumour size (cm)*			
⩽2	55 (17.5%)	259 (82.5%)	0.012
>2	68 (26.2%)	192 (73.8%)	
			
*pLymph node status*			
Negative	74 (18.7%)	321 (81.3%)	0.024
Positive	48 (27.1%)	129 (72.9%)	
			
*Lymph-vascular invasion*			
Negative	71 (18.5%)	313 (81.5%)	0.015
Positive	52 (27.4%)	138 (72.6%)	
			
*Histological type*			
IDC	108 (22.6%)	370 (77.4%)	0.129
Special type	15 (15.6%)	81 (84.4%)	

Abbreviations: TNBC=triple-negative breast cancer; IDC=invasive ductal carcinoma.

**Table 2:** Univariate and multivariate analyses with respect to overall survival in 574 all breast cancers

	**Univarite analysis**			**Multivariate analysis**		
**Parameters**	**Odds ratio**	**95% CI**	***P*-value**	**Odds ratio**	**95% CI**	***P*-value**
Pathological stage						
I *vs* II and III	2.199	0.987–4.898	0.049	1.122	0.360–3.495	0.842
						
Lymph node status						
Negative *vs* positive	3.083	1.541–6.169	0.001	2.341	0.873–6.281	0.091
						
Subtype						
TNBC *vs* non-TNBC	0.099	0.045–0.220	<0.001	0.144	0.061–0.341	<0.001
						
E-cadherin						
Negative *vs* positive	0.213	0.096–0.472	<0.001	0.517	0.218–1.230	0.136

Abbreviations: CI=confidence interval; TNBC=triple-negative breast cancer.

**Table 3:** Correlations between E-cadherin expression and clinicopathological parameters in 574 all breast cancers in 123 triple-negative breast cancers

	**All breast cancers (*n*=574)**			**TNBC (*n*=123)**		
**Parameters**	**Negative (*n*=238)**	**Positive (*n*=336)**	***P*-value**	**Negative (*n*=90)**	**Positive (*n*=33)**	***P*-value**
*ER and HER2 status*						
TNBC	90 (73.2%)	33 (26.8%)	<0.001	Not	Not	
Non-TNBC	148 (32.8%)	303 (67.2%)		determined	determined	
						
*Age at operation (years)*					1	
⩽55	100 (39.0%)	156 (61.0%)	0.295	40 (69.0%)	8 (31.0%)	0.320
>55	138 (43.4%)	180 (56.6%)		50 (77.0%)	15 (23.0%)	
						
*Pathological stage*						
I	100 (39.2%)	155 (60.8%)	0.328	29 (70.0%)	12 (30.0%)	0.666
II and III	138 (43.3%)	181 (56.7%)		61 (74.4%)	21 (25.6%)	
						
*pTumour size (cm)*						
⩽2	129 (41.1%)	185 (61.5%)	0.839	40 (72.7%)	15 (27.3%)	0.920
>2	109 (42.0%)	151 (58.0%)		50 (73.5%)	18 (26.5%)	
						
*pLymph node status*						
Negative	153 (38.5%)	244 (61.5%)	0.032	52 (69.3%)	23 (30.7%)	0.213
Positive	85 (48.0%)	92 (52.0%)		38 (79.2%)	10 (20.8%)	
						
*Lymph-vascular invasion*						
Negative	152 (39.6%)	232 (60.4%)	0.156	52 (74.3%)	18 (25.7%)	0.844
Positive	86 (45.3%)	104 (54.7%)		38 (71.7%)	15 (28.3%)	
						
*Histological type*						
IDC	193 (40.0%)	285 (60.0%)	0.238	77 (71.3%)	31 (28.7%)	0.208
Special type	45 (46.9%)	51 (53.1%)		13 (86.7%)	2 (13.3%)	

Abbreviations: ER=oestrogen receptor; HER2=human epidermal growth factor receptor 2; TNBC=triple-negative breast cancer; IDC=invasive ductal carcinoma.

**Table 4:** Univariate and multivariate analyses with respect to overall survival in 123 triple-negative breast cancers

	**Univarite analysis**			**Multivariate analysis**		
**Parameters**	**Odds ratio**	**95% CI**	***P*-value**	**Odds ratio**	**95% CI**	***P*-value**
*Pathological stage*						
I *vs* II and III	2.165	0.857–5.465	0.102	1.284	0.373–4.418	0.692
						
*Lymph node status*						
N0 *vs* N1–N3	2.421	1.114–5.262	0.026	1.794	0.652–4.936	0.258
						
*E-cadherin*						
Negative *vs* positive	0.197	0.047–0.836	0.028	0.227	0.053–0.971	0.046

Abbreviation: CI=confidence interval.
